# Vital signs of the systemic inflammatory response syndrome in adult patients with acute infections presenting in out-of-hours primary care: A cross-sectional study

**DOI:** 10.1080/13814788.2021.1917544

**Published:** 2021-05-12

**Authors:** Feike J. Loots, Daan Smulders, Paul Giesen, Rogier M. Hopstaken, Marleen Smits

**Affiliations:** a Scientific Centre for Quality of Healthcare (IQ healthcare), Radboud University Medical Centre, Radboud Institute for Health Sciences, Nijmegen, The Netherlands; b Julius Center for Health Sciences and Primary Care, University Medical Centre Utrecht, Utrecht, The Netherlands; c Star-Shl Diagnostic Centres, Etten-Leur, The Netherlands

**Keywords:** Systemic inflammatory response syndrome, vital signs, sepsis, general practice, after-hours care

## Abstract

**Background:**

Signs of the systemic inflammatory response syndrome (SIRS) – fever (or hypothermia), tachycardia and tachypnoea – are used in the hospital setting to identify patients with possible sepsis.

**Objectives:**

To determine how frequently abnormalities in the vital signs of SIRS are present in adult out-of-hours (OOH) primary care patients with suspected infections and assess the association with acute hospital referral.

**Methods:**

We conducted a cross-sectional study at the OOH GP cooperative in Nijmegen, the Netherlands, between August and October 2015. GPs were instructed to record the body temperature, heart rate and respiratory rate of all patients with suspected acute infections. Vital signs of SIRS, other relevant signs and symptoms, and referral state were extracted from the electronic registration system of the OOH GP cooperative retrospectively. Logistic regression analysis was used to evaluate the association between clinical signs and hospital referral.

**Results:**

A total of 558 patients with suspected infections were included. At least two SIRS vital signs were abnormal in 35/409 (8.6%) of the clinic consultations and 60/149 (40.3%) of the home visits. Referral rate increased from 13% when no SIRS vital sign was abnormal to 68% when all three SIRS vital signs were abnormal. Independent associations for referral were found for decreased oxygen saturation, hypotension and rapid illness progression, but not for individual SIRS vital signs.

**Conclusion:**

Although patients with abnormal vital signs of SIRS were referred more often, decreased oxygen saturation, hypotension and rapid illness progression seem to be most important for GPs to guide further management.

KEY MESSAGES

- Vital sign abnormalities of SIRS are present in 9% of adult patients with suspected infections during out-of-hours GP cooperative clinic consultations, compared to 40% in home visits.
- Rapid progression of illness, decreased peripheral oxygen saturation and hypotension are stronger associated with hospital referral than signs of SIRS.

## Introduction

Sepsis is a major cause of critical illness, with a global incidence of almost 50 million patients, resulting in 11 million deaths per year [1]. Early intravenous antibiotics administration is a central element, because mortality and morbidity resulting from sepsis increase after delayed treatment [2–4].

Most patients with acute infections are assessed in primary care initially, and timely referral of patients with sepsis to the emergency department (ED) by the general practitioner (GP) is essential to prevent unnecessary delay in treatment. A previous study by the authors’ research group has shown that one in three patients admitted to the intensive care unit due to community-acquired sepsis was not referred to a hospital by the GP after the first contact [5]. Furthermore, it has been found that sepsis was suspected in only a minority of patients who are referred, leading to non-urgent ambulance transports even in patients with septic shock [6,7].

In the past decades, systemic inflammatory response syndrome (SIRS) has been widely used to identify patients with sepsis [8]. SIRS is a syndrome characterised by two or more of the following symptoms: fever or hypothermia, tachycardia, tachypnoea and abnormal leucocyte count. Although a new international consensus definition of sepsis (Sepsis-3) was published in 2016 in which SIRS is no longer conditional for sepsis [9], screening for SIRS is still considered useful to identify patients at risk for sepsis [10,11]. Screening for symptoms of SIRS might also improve the recognition of sepsis in primary care. Although the leucocyte count is not readily available in primary care, the three vital signs of SIRS can be assessed during ambulance transport or triage in the ED.

As sepsis is an acute illness, patients often present out-of-hours (OOH). In the Netherlands, GPs are organised in GP cooperatives during OOH. These are often co-located with hospitals and usually serve catchment areas of between 100,000 and 400,000 inhabitants. In total, 119 GP cooperatives provide care to almost the entire Dutch population. Yearly about 250 contacts per 1000 inhabitants are performed, consisting of approximately 50% clinic consultations, 10% home visits and 40% telephone consultations [12].

This study’s objectives were to measure the presence of abnormalities in the vital signs of SIRS in adult patients with suspected infectious conditions who are assessed by GPs at OOH GP cooperatives, and assess the association with hospital referral.

## Methods

### Study design and setting

We conducted a cross-sectional study at one large GP cooperative, located in Nijmegen, the Netherlands. This GP cooperative delivers out-of-hours primary care to approximately 327,000 inhabitants, of whom about half live in the city of Nijmegen and half in the surrounding suburban to rural area. In 2015, 133,844 contacts were registered, consisting of 41% telephone consultations, 49% clinic consultations and 10% home visits [13].

### Procedure

During eight weeks between 30 August and 24 October 2015, a medical intern (DS) instructed attending GPs to measure the tympanic temperature, heart rate and respiratory rate in all patients in whom they had any suspicion of infection as the cause of the acute complaints and register the findings in the medical record. Although these measurements are standard care for patients with infections, not all GPs perform and record these in the patient file. Our efforts were, therefore, focused on motivating the GPs to minimise missing data on the SIRS parameters. The research team member personally explained the study to the attending GPs but did not assist the GPs during patient contacts. Small reminder cards and desktop clocks were also provided in all consultation rooms. In addition, chauffeurs assisting GPs during the home visits were instructed to remind the GP and help with the measurement of the vital signs. All patients received care as usual. The researcher was present to instruct the GPs in 28/40 weekday evenings and nights and 5/16 weekend days, accounting for 45.7% of the clinic consultations and home visits during the study period.

### Data collection

**Figure 1.**
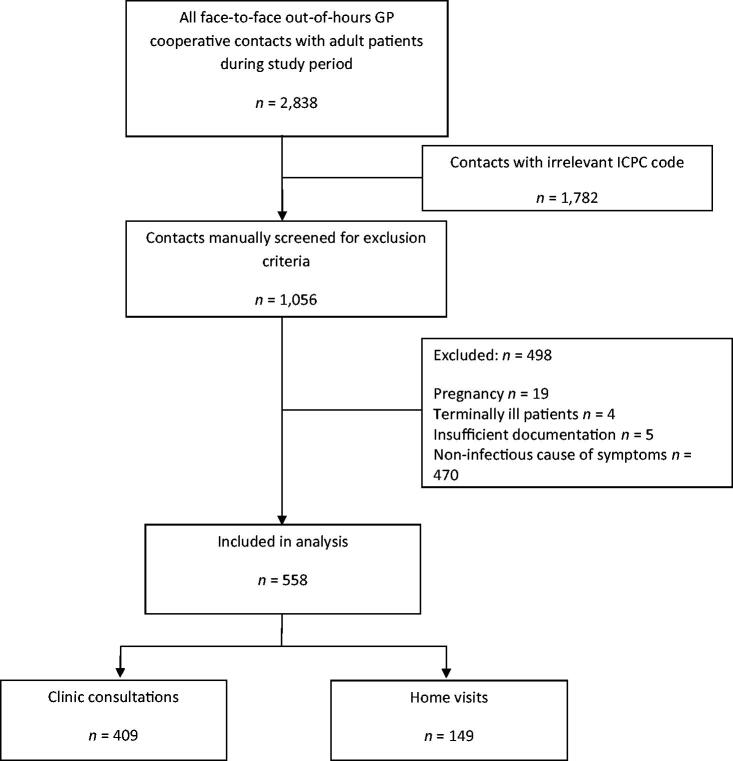
Flowchart of included patients.

Anonymised patient files of all clinic consultations and home visits in the study period were extracted from the GP cooperative registration system. Adult patients with suspected infection were eligible for inclusion. This concerned all acute infections, such as respiratory tract infections, abdominal infections, fever of unknown origin and localised infections (e.g. otitis or local abscess). Only contacts of GPs who received instruction (corresponding to the 45.7% of the clinic consultations and home visits as mentioned above) were included. Patients with more than one contact during the study period were analysed as separate index cases. ICPC (International Classification of Primary Care) codes that did not match (possible) infections were excluded during the extraction based on the codes listed in Supplemental Appendix 1. Next, all anonymised medical records were screened manually. Patients in whom the GP did not suspect an infection according to the differential diagnosis in the free text were excluded (e.g. trauma or renal colic). Other exclusion criteria were pregnant or terminally ill patients and records that were insufficiently documented to assess. Figure 1 shows a flowchart of included patients.

### Variables

The following variables were digitally extracted from the medical records: age and sex as background characteristics and (probable) diagnosis based on the ICPC code. We divided the included patients into the following groups, based on the ICPC codes (Supplemental Appendix 1): upper respiratory tract infections, lower respiratory tract infections, urogenital infections, abdominal infections, skin and soft tissue infections, fever of unknown origin and other infections. Other variables were manually retrieved from the free text of the medical records by a medical intern (DS). First, the vital signs of SIRS: temperature, heart rate and respiratory rate. SIRS criteria were defined as a temperature <36 or >38 °C, heart rate >90 beats/minute and respiratory rate >20 breaths/minute. Second, we also retrieved recording of the other relevant clinical signs and symptoms: systolic blood pressure, peripheral oxygen saturation (SpO_2_), shivering (yes/no), rapid progression of illness (yes/no), unable to walk normally (yes/no), altered mental status (yes/no). If a clinical symptom was not mentioned in the medical records, we considered it absent. In case the free text in the medical record was equivocal, the final decision on the presence or absence of a symptom was discussed with a general practitioner (PG) and an emergency physician (FL). Furthermore, antibiotic prescription (yes/no), hospital referral (yes/no) and 30-day mortality were retrieved from the GP cooperative registration system. We did not have access to data from patient’s GP or hospital data, as informed consent would be required.

### Data analyses

We used descriptive statistics for the background characteristics and clinical parameters of the study population. Mean and standard deviation (SD) were used for the description of normally distributed variables; median and interquartile range for non-normal distributions. We performed univariable and multivariable logistic regression analysis to examine the association of clinical signs and symptoms with hospital referral. Missing data of vital signs were assumed to be normal values. Still, for the analysis of the association between clinical signs and symptoms and hospital referral rate, we also performed a sensitivity analysis after imputing missing data using multiple imputation y chained equations [14]. Rubin’s rules were used to pool the results of 30 imputed data sets [15]. We used SPSS (IBM SPSS, version 25) for all data analyses.

### Ethics

We conducted the study in compliance with the declaration of Helsinki. The Ethical Research Committee of the Radboud University Medical Centre Nijmegen concluded that this study does not fall within the remit of the Dutch Medical Research Involving Human Subjects Act [Wet Mensgebonden Onderzoek] (file number 2016-2697).

## Results

A total of 2838 electronic medical records of clinic consultations and home visits in adult patients were retrieved. We selected 1056 patient records on relevant ICPC codes, which we screened manually for eligibility (Figure 1). In total, 558 patient records were included for analyses: 409 clinic consultations and 149 home visits.

Of the clinic consultations, 75/409 patients (18.3%) were referred to the hospital, and 45/149 (30.2%) of the patients receiving home visits were referred. Patients who received a home visit were older and more often presented themselves with lower respiratory tract infections than patients who received a clinic consultation. Temperature was the SIRS vital sign measured most often (84%). Heart rate and respiratory rate were measured, respectively, in 66% and 50% of the 558 included patients. In 95/558 (17%) of all patients, at least two SIRS vital signs were abnormal: 35/409 (8.6%) of the clinic consultations and 60/149 (40.3%) of the home visits. In total, five patients were recorded as deceased within 30 days after the initial contact with the GP cooperative. All five patients were visited at home, of whom three patients were not referred to the hospital (Table 1).

**Table 1.** Patient characteristics of the total study population and divided by contact type and by hospital referral.

Patient characteristic	All patients (*n* = 558)	Clinic consultations			Home visits		
		Not referred to hospital (*n* = 334)	Referred to hospital (*n* = 75)	Total (*n* = 409)	Not referred to hospital (*n* = 104)	Referred to hospital (*n* = 45)	Total (*n* = 149)
Background characteristics							
Age, median (IQR^a^), year	50 (31–71)	41 (27–55)	44 (27–61)	41 (27–56)	79 (68–84)	75(59–84)	78 (67–84)
Female, *N* (%)	315 (56.5)	196 (58.7)	40 (53.3)	236 (57.7)	57 (52.8)	22 (48.9)	79 (53.0)
Source of infection, *N* (%)							
Lower respiratory tract infection	102 (18.3)	47 (14.1)	3 (4.0)	50 (12.2)	36 (34.6)	16 (35.6)	52 (34.9)
Upper respiratory tract infection	78 (14.0)	65 (19.5)	7 (9.3)	72 (17.6)	6 (5.8)	0 (0.0)	6 (4.0)
Urogenital infection	107 (19.2)	73 (21.9)	3 (4.0)	76 (18.6)	26 (25.0)	5 (11.1)	31 (20.8)
Abdominal infection	95 (17.0)	38 (11.4)	39 (52.2)	77 (18.8)	8 (7.7)	9 (20.0)	18 (12.1)
Skin or soft tissue infection	103 (6.9)	89 (26.6)	5 (6.7)	94 (23.0)	8 (7.7)	1 (2.2)	9 (6.0)
Fever of unknown origin	48 (8.6)	8 (2.4)	14 (18.7)	22 (5.4)	16 (15.4)	10 (22.2)	26 (17.4)
Other	25 (4.5)	14 (4.2)	4 (5.3)	18 (4.4)	3 (2.9)	4 (8.9)	7 (4.7)
Vital signs of SIRS							
Body temperature measurement, *N* (%)	471 (84.4)	275 (82.3)	61 (81.3)	336 (82.2)	92 (88.5)	43 (95.6)	135 (90.7)
Body temperature, mean (SD^b^), °C	37.5 (0.9)	37.2 (0.7)	37.8 (1.0)	37.3 (0.8)	37.7 (1.0)	38.3 (1.1)	37.9 (1.1)
Respiratory rate measurement, *N* (%)	279 (50.0)	142 (42.5)	35 (46.7)	177 (43.3)	63 (60.6)	39 (88.7)	102 (68.5)
Respiratory rate, mean (SD), breaths/min	20 (6.9)	17 (4.7)	19 (6.0)	17 (5.1)	22 (6.5)	27 (7.5)	24 (7.3)
Heart rate measurement, *N* (%)	368 (65.9)	186 (55.7)	45 (60.0)	231 (56.5)	92 (88.5)	45 (100)	137 (91.9)
Heart rate, mean (SD), beats/min	87 (18.3)	83 (16.6)	91 (19.1)	85 (17.4)	87 (16.7)	100 (20.9)	92 (19.1)
≥2 SIRS vital sign abnormalities,^c^ *N* (%)	95 (17.0)	22 (6.6)	13 (17.3)	35 (8.6)	28 (26.9)	32 (71.1)	60 (40.3)
Antibiotics, *N* (%)	244 (43.7)	177 (53.0)	0 (0.0)	177 (43.3)	67 (64.4)	0 (0.0)	67 (45.0)
30-day mortality, *N* (%)	5 (0.9)	0 (0.0)	0 (0.0)	0 (0.0)	3 (2.9)	2 (4.4)	5 (3.4)

^a^Interquartile range.

^b^Standard deviation.

^c^Defined as body temperature <36 or >38 °C; tachycardia >90/min; tachypnoea >20/min.

In Table 2, differences in SIRS vital signs and other clinical signs and symptoms between patients who were and were not referred to the hospital are shown. With an increasing number of abnormalities of SIRS vital signs, the referral rate increased from 46/343 (13%) if none of the SIRS vital signs were abnormal, to 29/120 (24%) in cases with one, 22/61 (36%) with two, and 23/34 (68%) if all three SIRS vital signs were abnormal. However, none of the individual three SIRS vital signs showed a statistically significant independent association with hospital referral (Table 3). Age, shivering, altered mental status and inability to walk normally did not show an independent association with referral. For a rapid progression of illness and SpO_2_ <94% the association was highly significant (*p* < 0.001) with adjusted odds ratio (OR) of 3.75 (95% CI 2.26–6.20) for rapid illness progression and 5.23 (95% CI 2.40–11.4) for peripheral oxygen saturation. For hypotension (systolic blood pressure ≤100 mmHg), the adjusted OR was 4.07 (95% CI 1.16–14.3). A sensitivity analysis using multiple imputed data in which all vital signs were entered as continuous variables showed similar results. The only differences were for temperature (now independently associated with referral) and blood pressure (no independent association; Supplemental Appendix 2).

**Table 2.** Differences in patient characteristics between referred patients and patients not referred to the hospital.

Patient characteristic	Referred (*n* = 120)	Not referred (*n* = 438)
Age, median (IQR^a^), year	55 (35–75)	48 (31–69)
Vital signs of SIRS, *N* (%)		
Temperature <36 or >38 °C	48 (40)	66 (15)
Respiratory rate >20/min	44 (37)	55 (13)
Heart rate >90/min	50 (42)	81 (18)
Number of SIRS vital sign abnormalities, *N* (%)		
0 (*n* = 343)	46 (38)	297 (68)
1 (*n* = 120)	29 (24)	91 (21)
2 (*n* = 61)	22 (18)	39 (9)
3 (*n* = 34)	23 (19)	11 (3)
Other clinical signs and symptoms, *N* (%)		
Hypotension^b^	10 (8)	6 (1)
Peripheral oxygen saturation <94%	27 (23)	18 (4)
Shivering	37 (31)	68 (16)
Unable to walk normally	18 (15)	28 (6)
Rapid progression of illness	62 (52)	77 (18)
Altered mental status	9 (8)	12 (3)

^a^Interquartile range.

^b^Defined as systolic blood pressure ≤100 mmHg.

**Table 3.** Logistic regression analyses for association of patient characteristics with hospital referral: univariable and multivariable analyses.

	Univariable analyses		Multivariable analyses	
Patient characteristic	Odds ratio (95% CI^a^)	*p*-Value	Odds ratio (95% CI)	*p*-Value
Age (per year)	1.01 (1.00–1.02)	0.04	0.99 (0.98–1.00)	0.09
Vital signs of SIRS				
Temperature <36 or >38 °C	3.76 (2.40–5.89)	<0.001	1.68 (0.94–2.99)	0.08
Respiratory rate >20/min	4.03 (2.53–6.43)	<0.001	1.76 (0.94–3.30)	0.08
Heart rate >90/min	3.15 (2.04–4.87)	<0.001	1.36 (0.78–2.37)	0.3
Other clinical signs and symptoms (yes/no)				
Hypotension^b^	6.54 (2.33–18.4)	<0.001	4.07 (1.16–14.3)	0.03
Peripheral oxygen saturation <94%	6.77 (3.58–12.8)	<0.001	5.23 (2.40–11.4)	<0.001
Shivering	2.43 (1.52–3.87)	<0.001	1.12 (0.62–2.06)	0.7
Unable to walk normally	2.58 (1.38–4.96)	0.003	1.03 (0.46–2.28)	0.9
Rapid progression of illness	5.0 (3.25–7.74)	<0.001	3.75 (2.26–6.20)	<0.001
Altered mental status	2.88 (1.18–7.00)	0.02	1.50 (0.48–4.70)	0.5

^a^ Confidence interval; ^b^Defined as systolic blood pressure >100 mmHg.

## Discussion

### Main findings

In adult patients with suspected infection assessed in OOH primary care, we observed the vital signs of SIRS after instructing GPs to record the temperature, heart rate and respiratory rate systematically in these patients. In 40% of patients assessed during a home visit at least two SIRS vital signs were abnormal compared to 9% of clinic consultations. With an increasing number of abnormal SIRS vital signs, the referral rate increased from 13% if none were abnormal up to 68% if all three SIRS vital signs were abnormal. However, in this population, associations of the three individual SIRS criteria and hospital referral were not statistically significant. Of the other clinical signs and symptoms, only peripheral oxygen saturation was unequivocally associated with hospital referral. Furthermore, rapid progression of illness was associated independently with hospital referral, but not age, shivering, inability to walk normally or altered mental status.

### Strengths and limitations

A strength of the study is that all contacts were analysed during a study period in which GPs were instructed to measure the body temperature, respiratory rate and heart rate in all patients with suspected infection. Using this method, we obtained a complete count of all patients with suspected infections presenting in OOH primary care, and the abnormal SIRS vital signs in these patients. However, still, in more than half of the patients at least one of these measurements was missing. This implies that the true presence of abnormalities in the SIRS vital signs could be more frequent than shown in the data. We did not impute missing data for the primary analysis as missing data are more likely to be normal values (for example, in cases where temperature was not recorded by the GP, patients were unlikely to be febrile). Furthermore, GPs do not make their decision to refer patients based on unmeasured vital signs. However, the sensitivity analyses using multiple imputed data showed similar results, concluding that a significant bias has occurred due to missing data less likely.

Other limitations of the study are the data collection at a single GP cooperative in the Netherlands, and the relatively short study period in September–October. Results may differ in other locations or seasons. This study’s findings are not representative for the setting of primary care during office hours, as contacts are usually less urgent than in OOH.

### Comparison with existing literature

To the best of our knowledge, no previous research has been published assessing the presence of abnormalities in the SIRS vital signs in the primary care setting or relation with the referral rate. Tusgul et al. investigated the sensitivity of SIRS for adverse outcomes in patients with infections during ambulance transportation and at triage in the ED [16]. SIRS status was based on vital signs only and not on the leucocyte count. The reported rate of SIRS in the ambulance was 49% compared to 42% during triage in the ED in the same population. As the mortality rate was relatively low in patients with SIRS abnormalities who were referred after a home visit (30-day mortality of 4.4%), we do not suspect the patients in the current study to be more severely ill than the patients included in that paper (mortality of 3.7% at 48 h). In this study, SIRS was compared to the quick Sequential Organ Failure Assessment (qSOFA) score [17]. A qSOFA score ≥2 (of the items respiratory rate ≥ 22, systolic blood pressure < 100 mmHg and altered mental status) showed a poor sensitivity for adverse outcome and was present in only 19% of the study population during ambulance transport. We did not instruct GPs to record blood pressure and mental status in all patients, but retrieved this information if mentioned in the medical records. Both hypotension and altered mental status were present in only 8% of the referred patients.

### Implications for research and practice

In the Netherlands, guidelines for the management of sepsis by GPs are currently lacking. We do not advise implementing the SIRS screening tool to diagnose sepsis in primary care based on the current findings. Rather, the results should be interpreted as an indication of how often GPs are confronted with possible sepsis. This study shows this is relatively common, especially during OOH home visits to patients with suspected infections. Complete measurement of all vital signs during home visits of elderly patients with suspected infection can help GPs identify patients in early sepsis stages who do not appear critically ill otherwise. Not every patient who has abnormal SIRS vital signs needs hospital treatment. On the other hand, other clinical signs and symptoms – especially peripheral oxygen saturation and rapid progression of illness – appear to be more important for GPs than SIRS vital signs in subsequent referral. More research is needed to determine which vital signs are the most predictive of progression to sepsis and what clinically relevant cut-off values of vital signs are in the primary care setting to design a simple and effective screening tool. Rapid diagnostic tests such as CRP testing might add to the clinical decision-making process. Currently, our study group is performing a full, diagnostic study to develop a clinical decision rule with clinical signs and symptoms and including additional blood tests available at the point of care [18].

## Conclusion

Abnormalities in the SIRS vital signs in patients with suspected infections are relatively common in OOH primary care, especially in patients assessed during home visits. Although patients with abnormal vital signs of SIRS were more frequently referred to the hospital, decreased peripheral oxygen saturation, hypotension, and rapid progression of illness seem to be the most important clinical signs for GPs to guide further management.

## Supplementary Material

Supplemental Table

Supplemental Appendix 1
