# Supplementary material for: Vital signs of the systemic inflammatory response syndrome in adult patients with acute infections presenting in out-of-hours primary care: A cross-sectional study
Source: Eur J Gen Pract. 2021 May 12;27(1):83–9. doi: 10.1080/13814788.2021.1917544 (PMC8118397; doi:10.1080/13814788.2021.1917544)
Supplement: Supplemental Table [file IGEN_A_1917544_SM6246.docx]

|  | | Univariable analyses | | Multivariable analyses | |
| --- | --- | --- | --- | --- | --- |
| Patient characteristic | | Odds ratio (95% CI^a^) | *P*-value | Odds ratio (95% CI) | *P*-value |
| Age (per year) | | 1.01 (1.00-1.02) | 0.043 | 0.99 (0.97-1.00) | 0.04 |
| Vital signs of SIRS | |  |  |  |  |
|  | Temperature (per °C) | 2.12 (1.68-2.69) | < 0.001 | 1.53 (1.11-2.11) | 0.01 |
|  | Respiratory rate (per breath/ min) | 1.09 (1.05-1.13) | < 0.001 | 1.04 (0.98-1.09) | 0.2 |
|  | Heart rate (per beat/min) | 1.03 (1.02-1.04) | < 0.001 | 1.01 (0.99-1.03) | 0.4 |
| Other clinical signs and symptoms | |  |  |  |  |
|  | Systolic blood pressure (per mmHg) | 0.99 (0.98-1.00) | 0.08 | 0.99 (0.98-1.01) | 0.3 |
|  | SpO_2_^b^ (per %) | 0.81 (0.74-0.88) | < 0.001 | 0.82 (0.72-0.92) | 0.001 |
|  | Shivering | 2.42 (1.52-3.87) | < 0.001 | 1.20 (0.65-2.22) | 0.6 |
|  | Unable to walk normally | 2.58 (1.38-4.86) | 0.003 | 1.24 (0.56-2.73) | 0.6 |
|  | Rapid progression of illness | 5.01 (3.27-7.74) | < 0.001 | 3.57 (2.10-6.06) | < 0.001 |
|  | Altered mental status | 2.88 (1.19-7.00) | 0.02 | 1.57 (0.50-4.91) | 0.4 |

Logistic regression analysis based on multiple imputed data with vital signs as continuous variables. Univariable and multivariable association with hospital referral.

^a^ Confidence Interval. ^b^ Peripheral oxygen saturation.
