# Supplementary material for: Vital signs of the systemic inflammatory response syndrome in adult patients with acute infections presenting in out-of-hours primary care: A cross-sectional study
Source: Eur J Gen Pract. 2021 May 12;27(1):83–9. doi: 10.1080/13814788.2021.1917544 (PMC8118397; doi:10.1080/13814788.2021.1917544)
Supplement: Supplemental Appendix 1 [file IGEN_A_1917544_SM6245.pdf]

Appendix 1.

Codes of the International Classification of Primary Care (2nd Edition) extracted for further manual review.

A01 Pain general/multiple sites
A02 Chills
A03 Fever
A04 Weakness/tiredness general
A05 Feeling ill
A06 Fainting/syncope
A29 General symptom/complaint other
A75 Infectious mononucleosis
A76 Viral exanthem other
A77 Viral disease other/NOS
A78 Infectious disease other/NOS
A99 General disease NOS
B02 Lymph gland(s) enlarged/painful
B70 Lymphadenitis acute
B99 Blood/lymph/spleen disease other
D01 Abdominal pain/cramps general
D02 Abdominal pain epigastric
D06 Abdominal pain localized other
D09 Nausea
D10 Vomiting
D11 Diarrhoea
D18 Change faeces/bowel movements
D25 Abdominal distension
D29 Digestive symptom/complaint other
D70 Gastrointestinal infection
D73 Gastroenteritis presumed infection
D83 Mouth/tongue/lip disease
D88 Appendicitis
D92 Diverticular disease
D95 Anal fissure/perianal abscess
D98 Cholecystitis/cholelithiasis
D99 Disease digestive system, other
H70 Otitis externa
H71 Acute otitis media/myringitis
H72 Serous otitis media
K70 Infection of circulatory system
K94 Phlebitis/thrombophlebitis
L04 Chest symptom/complaint
L05 Flank/axilla symptom/complaint
L12 Hand/finger symptom/complaint
L14 Leg/thigh symptom/complaint
L20 Joint symptom/complaint NOS
L29 Sympt/complt. Musculoskeletal other
L70 Infections musculoskeletal system
L99 Musculoskeletal disease, other
N01 Headache
N18 Paralysis/weakness
N71 Meningitis/encephalitis
N73 Neurological infection other
R01 Pain respiratory system
R02 Shortness of breath/dyspnoea
R04 Breathing problem, other
R05 Cough
R09 Sinus symptom/complaint
R21 Throat symptom/complaint
R24 Haemoptysis
R25 Sputum/phlegm abnormal
R29 Respiratory symptom/complaint oth.
R71 Whooping cough
R72 Strep throat
R74 Upper respiratory infection acute
R75 Sinusitis acute/chronic
R76 Tonsillitis acute
R77 Laryngitis/tracheitis acute
R78 Acute bronchitis/bronchiolitis
R80 Influenza
R81 Pneumonia
R82 Pleurisy/pleural effusion
R83 Respiratory infection other
R95 Chronic obstructive pulmonary dis
R99 Respiratory disease other
S01 Pain/tenderness of skin
S06 Rash localized
S07 Rash generalized
S08 Skin colour change
S09 Infected finger/toe
S10 Boil/carbuncle
S11 Skin infection post-traumatic
S76 Skin infection other
T11 Dehydration
T70 Endocrine infection
U01 Dysuria/painful urination
U02 Urinary frequency/urgency
U14 Kidney symptom/complaint
U70 Pyelonephritis/pyelitis
U71 Cystitis/urinary infection other
U72 Urethritis
U98 Abnormal urine test NOS
U99 Urinary disease, other
W70 Puerperal infection/sepsis
W94 Puerperal mastitis
X01 Genital pain female
X04 Painful intercourse female
X14 Vaginal discharge
X17 Pelvis symptom/complaint female
X71 Gonorrhoea female
X74 Pelvic inflammatory disease
X84 Vaginitis/vulvitis NOS
X85 Cervical disease NOS
Y06 Prostate symptom/complaint
Y71 Gonorrhoea male
Y73 Prostatitis/seminal vesiculitis
Y74 Orchitis/epididymitis
Y75 Balanitis
